# Supplementary material for: Comparison of mental health and burnout between medical and nonmedical students
Source: PLoS One. 2025 Oct 9;20(10):e0328145. doi: 10.1371/journal.pone.0328145 (PMC12510498; doi:10.1371/journal.pone.0328145)
Supplement: S1 Table — (PDF) [file pone.0328145.s001.pdf]

S1 Table. T-tests comparing medical students to nonmedical students based on complete cases

	Overall sample (N=1963)			Medical students (N = 1093)		Nonmedical students (N = 870)		t-tests			
	%Missing	Mean	SD	Mean	SD	Mean	SD	N	t	p	Cohen's d
**Mental Health**											
Depressive symptoms	8.15	20.16	11.63	18.99	11.35	21.90	11.84	1803	5.25	.000	0.25
Suicidal ideation	8.41	0.88	1.20	0.65	1.03	1.23	1.35	1798	10.40	.000	0.50
Anxiety symptoms	9.02	47.56	11.97	45.98	11.92	49.97	11.65	1786	6.97	.000	0.34
**Burnout**											
Emotional exhaustion	9.32	16.56	5.19	16.50	4.91	16.66	5.59	1780	0.64	.521	0.03
Cynicism	9.32	10.22	4.65	9.48	4.29	11.36	4.95	1780	8.50	.000	0.41
Academic efficacy	9.32	23.94	4.67	24.00	4.53	23.85	4.90	1780	-0.64	.520	0.03
**Risk factors**											
Identifying as male	0.00	0.28	0.45	0.32	0.47	0.23	0.42	1963	-4.53	.000	0.20
Age	0.10	22.56	3.76	22.09	3.03	23.14	4.46	1961	6.16	.000	0.28
Material deprivation	1.32	1.13	1.55	1.01	1.43	1.30	1.68	1937	4.09	.000	0.19
Health deprivation	1.88	0.30	0.56	0.23	0.51	0.40	0.62	1926	6.76	.000	0.30
Sleep hours per day	0.25	7.12	0.94	7.03	0.92	7.22	0.97	1958	4.41	.000	0.20
Physical activities	0.25	3.42	2.94	3.51	2.86	3.31	3.02	1958	-1.49	.000	0.07
Satisfaction with health	0.25	3.61	0.99	3.73	0.97	3.45	0.99	1958	-6.21	.000	0.29
Emotion-focused coping	9.37	10.12	4.14	9.57	3.99	10.96	4.23	1779	6.98	.000	0.34
Problem-focused coping	9.37	7.17	1.77	7.27	1.69	7.02	1.87	1779	-2.99	.003	0.14
Help-seeking coping	9.48	5.33	2.83	5.57	2.88	4.96	2.71	1777	-4.46	.000	0.22
Hours in paid job per week	0.15	4.22	7.53	2.96	6.62	5.79	8.28	1960	8.44	.000	0.38
Social deprivation	1.88	0.50	0.76	0.45	0.70	0.57	0.81	1926	3.54	.000	0.16
Emotional social support	9.48	8.48	2.04	8.62	1.98	8.27	2.12	1777	-3.51	.001	0.17
Practical social support	9.48	7.67	2.32	7.83	2.31	7.43	2.32	1777	-3.54	.000	0.17

Note. Cohen's *d*s of 0.2, 0.5, and 0.8 are considered respectively as small, medium, and large [36].
