# Supplementary material for: Comparison of mental health and burnout between medical and nonmedical students
Source: PLoS One. 2025 Oct 9;20(10):e0328145. doi: 10.1371/journal.pone.0328145 (PMC12510498; doi:10.1371/journal.pone.0328145)
Supplement: S2 Table — (PDF) [file pone.0328145.s002.pdf]

S2 Table. Adjusted regressions testing the difference between medical and nonmedical students based on complete cases (N = 1,777)

	Mental health									Burnout								
	Depressive symptoms			*Suicidal ideation*			*Anxiety symptoms*			*Emotional exhaustion*			*Cynicism*			*Academic efficacy*		
	β	*SE*	*p*	*β*	*SE*	*p*	*β*	*SE*	*p*	*β*	*SE*	*p*	*β*	*SE*	*p*	*β*	*SE*	*p*
**Medical students**	.04	0.41	.040	-.12	0.05	<.001	-.01	0.41	.533	.10	0.23	<.001	-.13	0.22	<.001	-.07	0.22	.001
Identifying as male	-.06	0.44	<.001	.02	0.06	.297	-.07	0.46	<.001	-.05	0.27	.028	.06	0.25	.022	-.03	0.25	.186
Curriculum year	-.09	0.12	<.001	-.04	0.01	.073	-.06	0.12	<.001	-.07	0.07	.003	.15	0.07	<.001	-.02	0.06	.332
Material deprivation	.07	0.14	.001	.02	0.02	.470	.03	0.13	.094	.06	0.08	.012	.04	0.08	.117	.00	0.07	.874
Health deprivation	.19	0.48	<.001	.20	0.06	<.001	.14	0.42	<.001	.13	0.21	<.001	.06	0.23	.021	-.05	0.21	.030
Sleep hours per day	-.11	0.21	<.001	-.05	0.03	.022	-.10	0.21	<.001	-.11	0.12	<.001	-.01	0.12	.733	.05	0.11	.018
Physical activities	.00	0.07	.941	.00	0.01	.945	-.01	0.07	.487	-.07	0.04	.001	.01	0.04	.752	.03	0.04	.175
Satisfaction with health	-.18	0.23	<.001	-.10	0.03	<.001	-.16	0.23	<.001	-.14	0.13	<.001	-.12	0.12	<.001	.11	0.12	<.001
Emotion-focused coping	.39	0.06	<.001	.24	0.01	<.001	.49	0.05	<.001	.31	0.03	<.001	.25	0.03	<.001	-.25	0.03	<.001
Problem-focused coping	.01	0.11	.585	-.02	0.01	.255	-.01	0.11	.399	-.01	0.07	.822	-.06	0.06	.009	.18	0.07	<.001
Help-seeking coping	-.08	0.08	<.001	-.08	0.01	<.001	-.05	0.08	.009	-.04	0.04	.138	-.07	0.04	.006	.08	0.04	.001
Hours in paid job per week	.00	0.03	.979	.01	0.00	.658	.00	0.03	.803	.00	0.01	.984	.04	0.01	.117	-.02	0.01	.487
Social deprivation	.06	0.29	.002	.07	0.04	.001	.02	0.29	.164	.06	0.17	.014	-.02	0.16	.378	.02	0.17	.410
Emotional social support	-.06	0.12	.005	-.06	0.02	.060	-.04	0.12	.073	.02	0.07	.575	-.06	0.07	.060	.08	0.07	.014
Practical social support	-.11	0.11	<.001	-.11	0.02	<.001	-.11	0.11	<.001	-.05	0.06	.072	-.02	0.06	.554	.10	0.06	.002
F	145.00			48.04			187.60			56.73			29.05			33.81		
F's p-value	<.001			<.001			<.001			<.001			<.001			<.001		
R2	.55			.33			.57			.32			.20			.22		
