# Supplementary material for: Comparison of mental health and burnout between medical and nonmedical students
Source: PLoS One. 2025 Oct 9;20(10):e0328145. doi: 10.1371/journal.pone.0328145 (PMC12510498; doi:10.1371/journal.pone.0328145)
Supplement: S3 Table — (PDF) [file pone.0328145.s003.pdf]

S3 Table. T-tests comparing medical students to nonmedical students while excluding sixth-year medical students

	Overall sample (N=1827)			Medical students (N = 957)		Nonmedical students (N = 870)		t-tests (N after MI = 1926)		
	%Missing	Mean	SD	Mean	SD	Mean	SD	t	p	Cohen's d
**Mental Health**										
Depressive symptoms	8.70	20.68	11.62	19.74	11.37	21.90	11.84	-3.88	.000	0.19
Suicidal ideation	8.98	0.92	1.23	0.68	1.06	1.23	1.35	-9.33	.000	0.46
Anxiety symptoms	9.63	48.01	11.88	46.55	11.84	49.97	11.65	-6.08	.000	0.29
**Burnout**										
Emotional exhaustion	9.96	16.66	5.18	16.65	4.85	16.66	5.59	-0.15	.881	0.00
Cynicism	9.96	10.24	4.67	9.40	4.27	11.36	4.95	-8.80	.000	0.43
Academic efficacy	9.96	23.86	4.70	23.87	4.55	23.85	4.90	0.22	.827	0.00
**Risk factors**										
Identifying as male	0.00	0.28	0.45	0.32	0.47	0.23	0.42	4.55	.000	0.21
Age	0.05	22.36	3.75	21.66	2.79	23.14	4.46	-8.60	.000	0.40
Material deprivation	1.42	1.14	1.55	0.99	1.41	1.30	1.68	-4.18	.000	0.20
Health deprivation	2.03	0.31	0.57	0.23	0.52	0.40	0.62	-6.16	.000	0.29
Sleep hours per day	0.27	7.12	0.96	7.03	0.94	7.22	0.97	-4.26	.000	0.20
Physical activities	0.27	3.40	2.96	3.49	2.89	3.31	3.02	1.29	.199	0.06
Satisfaction with health	0.27	3.58	0.99	3.70	0.97	3.45	0.99	5.39	.000	0.25
Emotion-focused coping	10.02	10.25	4.16	9.73	4.03	10.96	4.23	-6.13	.000	0.30
Problem-focused coping	10.02	7.14	1.78	7.24	1.71	7.02	1.87	2.44	.015	0.12
Help-seeking coping	10.13	5.22	2.82	5.42	2.88	4.96	2.71	3.48	.001	0.16
Hours in paid job per week	0.16	4.01	6.77	2.38	4.42	5.79	8.28	-11.14	.000	0.52
Social deprivation	2.03	0.51	0.76	0.46	0.71	0.57	0.81	-3.10	.002	0.14
Emotional social support	10.13	8.44	2.06	8.57	2.00	8.27	2.12	2.97	.003	0.15
Practical social support	10.13	7.60	2.34	7.73	2.34	7.43	2.32	2.65	.008	0.13

Note. MI = Multiple imputation. Cohen's *d*s of 0.2, 0.5, and 0.8 are considered respectively as small, medium, and large [36].
