# Supplementary material for: Comparison of mental health and burnout between medical and nonmedical students
Source: PLoS One. 2025 Oct 9;20(10):e0328145. doi: 10.1371/journal.pone.0328145 (PMC12510498; doi:10.1371/journal.pone.0328145)
Supplement: S4 Table — (PDF) [file pone.0328145.s004.pdf]

S4 Table. Adjusted regressions testing the difference between medical and nonmedical students while excluding sixth-year medical students (N=1790)

	Mental health									Burnout								
	Depressive symptoms			*Suicidal ideation*			*Anxiety symptoms*			*Emotional exhaustion*			*Cynicism*			*Academic efficacy*		
	β	*SE*	*p*	*β*	*SE*	*p*	*β*	*SE*	*p*	*β*	*SE*	*p*	*β*	*SE*	*p*	*β*	*SE*	*p*
**Medical students**	.04	0.43	.043	-.12	0.05	<.001	-.02	0.42	.291	.09	0.23	<.001	-.13	0.23	<.001	-.07	0.23	.003
Identifying as male	-.06	0.48	.001	.03	0.06	.150	-.07	0.47	<.001	-.05	0.27	.027	.05	0.26	.036	-.04	0.26	.118
Curriculum year	-.07	0.14	<.001	-.04	0.02	.041	-.06	0.14	.002	-.06	0.08	.004	.13	0.08	<.001	-.03	0.07	.274
Material deprivation	.07	0.15	<.001	.02	0.02	.341	.03	0.13	.088	.06	0.08	.016	.05	0.09	.080	.00	0.08	.858
Health deprivation	.19	0.47	<.001	.20	0.06	<.001	.14	0.41	<.001	.14	0.21	<.001	.07	0.22	.014	-.07	0.21	.005
Sleep hours per day	-.10	0.21	<.001	-.04	0.03	.037	-.10	0.21	<.001	-.10	0.12	<.001	.00	0.12	.960	.05	0.12	.022
Physical activities	.00	0.07	.812	.00	0.01	.934	-.01	0.07	.509	-.07	0.04	.001	.01	0.04	.778	.04	0.04	.131
Satisfaction with health	-.18	0.23	<.001	-.09	0.03	<.001	-.16	0.24	<.001	-.14	0.13	<.001	-.12	0.13	<.001	.10	0.12	<.001
Emotion-focused coping	.39	0.06	<.001	.23	0.01	<.001	.49	0.06	<.001	.31	0.03	<.001	.24	0.03	<.001	-.23	0.03	<.001
Problem-focused coping	.01	0.12	.784	-.03	0.02	.205	-.02	0.12	.297	-.01	0.07	.536	-.07	0.06	.002	.19	0.07	<.001
Help-seeking coping	-.08	0.08	<.001	-.09	0.01	<.001	-.05	0.08	.009	-.04	0.04	.126	-.07	0.04	.005	.08	0.04	.002
Hours in paid job per week	-.01	0.03	.458	.01	0.00	.562	-.03	0.03	.134	-.02	0.02	.354	.03	0.02	.218	.00	0.02	.979
Social deprivation	.05	0.29	.006	.07	0.04	.002	.02	0.29	.225	.06	0.17	.019	-.02	0.16	.387	.02	0.17	.409
Emotional social support	-.07	0.13	.003	-.07	0.02	.028	-.04	0.13	.066	.01	0.07	.620	-.07	0.07	.028	.09	0.07	.007
Practical social support	-.10	0.11	<.001	-.11	0.02	.001	-.11	0.11	<.001	-.05	0.07	.073	-.02	0.06	.610	.10	0.06	.003
F	131.38			47.35			167.07			53.96			28.21			31.89		
F's p-value	<.001			<.001			<.001			<.001			<.001			<.001		
R2	.54			.33			.56			.32			.20			.22		
